# Water and sanitation infrastructure for health: The impact of foreign aid

**DOI:** 10.1186/1744-8603-6-12

**Published:** 2010-07-29

**Authors:** Marianne J Botting, Edoye O Porbeni, Michel R Joffres, Bradley C Johnston, Robert E Black, Edward J Mills

**Affiliations:** 1 Sunnybrook Health Sciences Centre, University of Toronto, Toronto, Canada; 2 Faculty of Health Sciences, Simon Fraser University, Burnaby, Canada; 3 Department of Clinical Epidemiology and Biostatistics, McMaster University, Hamilton, Canada; 4 Department of International Health, Johns Hopkins Bloomberg School of Public Health, Johns Hopkins University, Baltimore, USA; 5 Interdisciplinary School of Health Sciences, Faculty of Health Sciences, University of Ottawa, Ottawa, Canada

## Abstract

**Background:**

The accessibility to improved water and sanitation has been understood as a crucial mechanism to save infants and children from the adverse health outcomes associated with diarrheal disease. This knowledge stimulated the worldwide donor community to develop a specific category of aid aimed at the water and sanitation sector. The actual impact of this assistance on increasing population access to improved water and sanitation and reducing child mortality has not been examined.

**Methods:**

We performed a country-level analysis of the relationship between water and sanitation designated official development assistance (WSS-ODA) per capita, water and sanitation coverage, and infant and child mortality in low-income countries as defined by the World Bank. We focused our inquiry to aid effectiveness since the establishment of the Millennium Development Goals (MDGs).

**Results:**

Access to improved water has consistently improved since 2002. Countries receiving the most WSS-ODA ranged from odds ratios of 4 to 18 times more likely than countries in the lowest tertile of assistance to achieve greater gains in population access to improved water supply. However, while there were modestly increased odds of sanitation access, these were largely non-significant. The countries with greatest gains in sanitation were 8-9 times more likely to have greater reductions in infant and child mortality.

**Conclusions:**

Official development assistance is importantly impacting access to safe water, yet access to improved sanitation remains poor. This highlights the need for decision-makers to be more intentional with allocating WSS-ODA towards sanitation projects.

## Background

Worldwide, 18% of all deaths in children under five are due to diarrheal diseases, accounting for approximately 1.4 million deaths per year. This makes diarrheal diseases a leading cause of child death globally[1,2]. The most common cause of diarrheal diseases results from gastrointestinal infections[3,4]. The majority of diarrheal deaths in children are due to the loss of large quantities of water and electrolytes (sodium, chloride and potassium) through liquid stool, resulting in severe dehydration and acidosis[5].

Since diarrheal diseases are primarily spread through the faecal-oral route, preventive measures include improving access to safe drinking water and adequate sanitation. Wealthy nations and international bodies first began designating assistance for water and sanitation specifically through the World Bank in 1961 [6]. The history of development assistance in the water and sanitation sector, summarized by Grover and others, includes investment in service provision and infrastructure, and is marked by numerous international conferences and declarations, multilateral organizational involvement, the International Drinking Water Supply and Sanitation Decade (1990s), and the creation of water working groups, councils, and partnerships [6-11]. In 2000, the Millennium Development Goals (MDGs), were developed as a way to draw attention to global health and social justice issues and measure global progress on these goals. Target four under Goal 7 is to "halve, by 2015, the proportion of the population without sustainable access to safe drinking water and basic sanitation"[12]. Goal 4 is to "Reduce by two-thirds, the under-five mortality rate". The adoption of the MDGs may in part explain the increase in overseas development assistance (ODA) to over 5 times that of 1990 levels[13].

Studies on aid effectiveness have been mixed. Most have dealt with the relationship between ODA and economic growth[14-16] the effect of predictability[17] and aid modality[18,19] on development. More recently some have examined the effectiveness of foreign aid in poverty reduction and human development [20-22]. Only one study has looked at aid effectiveness and population access to water and sanitation, though as part of a framework examining public service delivery in general [23]. Our aim was to specifically examine the relationship between per capita ODA designated to the water and sanitation, the change in population access to improved water and sanitation services, and subsequent indicators of child health.

## Methods

### Study Design and Rationale

Our study is a country-level analysis of the relationship between disbursements of official development assistance (ODA) per capita, improved water and sanitation coverage, and infant and child mortality since the establishment of the MDGs. Disbursed ODA was chosen since promised ODA has not yet had the chance to effect change. Countries included in this analysis were the 49 low-income economies of the world as defined by the World Bank [24]. Nearly 70 percent of the countries are in Africa. The low-income country category was chosen because of expected low levels of water and sanitation-related infrastructure and high influx of ODA.

### Data Collection

All included countries had data for water and sanitation access and ODA. All ODA statistics for the years 2002-2006 were sourced from the Organization for Economic Cooperation and Development Creditor Reporting System database [25]. Data on coverage of safe water and sanitation for the MDGs was gathered from The official United Nations site for the MDG indicators for 2000 and 2006 [26]. These data come from the WHO/UNICEF Joint Monitoring Programme, which has specific definitions for improved water supply and sanitation facilities. An improved water supply is defined as any of the following sources: piped water into a dwelling, plot, or yard; public tap or standpipe; tubewell or borehole; protected dug well; protected spring; or rainwater. Options that qualify as improved sanitation are: flush or pour-flush toilets connected to a sewer or septic tank, pit latrines, Ventilated Improved pit latrines, pit latrines with a slab, and composting toilets. It should be noted that since 2000, the Joint Monitoring Programme has used multiple population-based surveys rather than estimates of coverage by service providers, and values are derived from regression analysis to give the best estimate of coverage in a single year [27]. Infant mortality rate (IMR) and child mortality rate (CMR) figures were sourced from the World Health Organization Statistical Information System (WHOSIS) [27]. The IMR and CMR data were gathered for the years 2000 and 2006. The IMR and CMR indicators were chosen for child health outcomes due to the lack of both baseline (year 2000 or before) and more recent (after year 2000) data points for diarrhoeal-specific death rates.

We gathered information on potential confounders and effect modifiers from various sources. Country population, gross domestic product (GDP) and health expenditure statistics are sourced from WHOSIS [27]. For population and GDP, the latest available statistics are used. Health expenditure data was collected for the years 2000-2006 for all countries except Laos and Somalia. We sourced Corruption Perception Index data for 43 of the countries in our sample from the Transparency International annual survey for 2006 [28]. The index uses a scale of one to ten, with one being the most corrupt. We collected data on land area statistics for all 49 countries from the US Central Intelligence Agency World Factbook. Adjusting variables were included in the regression modelling and odds ratio calculations, as specified in the data tables.

### Statistical Analysis

We calculated the change in access to improved water and sanitation as the difference in percent coverage between 2000 and 2006. Sao Tomé and Principe was excluded from the analysis due to an atypically high influx of ODA in 2002 and 2003, which made the ODA per capita out of the range of the other countries due to their small populations.

Two values of change in outcomes (water coverage, sanitation coverage, IMR, and CMR) were calculated, namely absolute change and relative change. The absolute change was calculated simply by subtracting the value in 2000 from the value in 2006. The relative change was calculated by taking the absolute change and dividing by the 2000 baseline value. Unless otherwise stated, the values presented are relative change.

Variables were assessed for normality, and found in general to have skewed distributions. Thus, Spearman rank correlation coefficients were obtained to identify statistically significant relationships between variables. To assess the associations between variables of interest, unadjusted and adjusted odds ratios and 95% confidence intervals were estimated by unconditional logistic regression. The Mantel-Haenszel Statistic and the Breslow-Day test for homogeneity of the odds ratio were used to assess potential confounding. Using these results, we adjusted for area and country population using logistic regression. We used 2-sided p-values and all p-values are exact. All statistical analysis was performed using Statistical Analysis Software (SAS) 9.1.

Here it should be noted that the mismatch in years between ODA and outcomes (water and sanitation coverage, and IMR/CMR), though not ideal, does not negate the findings of this analysis. The year 2000 was the closest year available to the beginning of the ODA data for outcome variables, and thus is considered as a baseline value. Analysis focuses on the absolute or relative change in outcomes in relation to ODA flows. All years of ODA are compared individually to the change in outcomes between 2000 and 2006 to attempt to quantify the average lag in effect between ODA delivery and change in outcome.

## Results

### Sample characteristics

Countries varied greatly in land area, and in total water and sanitation designated official development assistance (WSS-ODA) received, as evidenced by the differences between medians and their corresponding means. In general, WSS-ODA has risen steadily between 2002 and 2006. Overall increases in water and sanitation coverage alongside decreases in IMR and CMR were observed between 2000 and 2006. A summary of data for collected variables is displayed in Table 1.

**Table 1:** Summary statistics for key country characteristics

	Median	Mean	Standard Error	n
Land area (km^2^)	259,828.50	444,992.79	66,057.82	48
Gross Domestic Product ($PPP)	1,120.00	1,144.78	82.68	46
Sum of all ODA from 2002 to 2006 (millions $USD)	1,156.68	2,191.46	434.28	48
Per capita WSS-ODA ($USD)				
2002-2006	2.73	3.41	0.45	48
2002	0.25	0.42	0.06	47
2003	0.35	0.60	0.09	49
2004	0.44	0.66	0.11	48
2005	0.53	0.80	0.13	48
2006	0.59	0.94	0.13	48
Change in % access to safe water between 2000 and 2006	4.76	9.80	2.09	48
Change in % access to safe sanitation between 2000 and 2006	9.09	16.22	3.42	47
% change in infant mortality rate between 2000 and 2006	-8.66	-10.39	1.38	48
% change in child mortality rate between 2000 and 2006	-9.64	-11.68	1.58	48
Corruption Perception Index 2006	2.40	2.52	0.08	43
Per capita government health expenditure 2006 ($USD)	25.00	34.65	4.23	48

PPP: Purchasing Power Parity

ODA: Official Development Assistance

WSS-ODA: Water and sanitation sector designated official development assistance

Corruption Perception Index uses a scale of 1 to 10; corruption is highest at level 1

### Correlations

Statistically significant correlations (Table 2) were observed for all years of WSS-ODA per capita and the change in water access except for 2005 and 2006, with the strongest correlation occurring for ODA given in 2004 (p = 0.004). Interestingly, the change in access to sanitation was negatively associated with the per capita government health expenditure in 2006 (p = 0.025).

**Table 2:** Spearman's rank correlation coefficients between selected variables

First Variable	Second Variable	Spearman Correlation	p	n
Change in % access to safe water	Per capita WSS-ODA 2002-2006	0.35	0.014*	48
	Per capita WSS-ODA 2002	0.33	0.024*	47
	Per capita WSS-ODA 2003	0.38	0.008*	48
	Per capita WSS-ODA 2004	0.41	0.004*	48
	Per capita WSS-ODA 2005	0.27	0.067	48
	Per capita WSS-ODA 2006	0.24	0.106	48
				
	Relative % change in access to improved sanitation	0.42	0.003*	47
				
	Relative % change IMR^†^	-0.08	0.592	48
	Relative % change CMR^†^	-0.06	0.688	48
				
Change in % access to improved sanitation	Per capita WSS-ODA 2002-2006	0.17	0.252	47
	Per capita WSS-ODA 2002	0.22	0.148	46
	Per capita WSS-ODA 2003	0.21	0.148	47
	Per capita WSS-ODA 2004	0.17	0.261	47
	Per capita WSS-ODA 2005	0.08	0.585	47
	Per capita WSS-ODA 2006	0.08	0.608	47
				
	Per capita government health expenditure 2006	-0.32	0.025*	47
				
	Relative % change IMR^†^	-0.19	0.186	47
	Relative % change CMR^†^	-0.23	0.117	47

*: Statistically significant at the alpha = 0.05 level

^†^: Correlated with absolute, and not relative change in % access

WSS-ODA: Water and sanitation designated official development assistance

IMR: Infant mortality rate

CMR: Child mortality rate

In cases where no correlation was observed, we cannot conclude that there is indeed no true association due to the limitation on statistical power determined by the small sample size of the analysis. Hence it is with this disclaimer that we report that our analysis did not detect statistically significant correlations between total levels of ODA and any health or infrastructure changes; absolute change in water access and child health; WSS-ODA and changes in access to improved sanitation services; and finally country GDP and absolute change in access to improved water supply.

### Aid and access

Table 3 summarizes the odds of increasing access to safe water and sanitation by the amount observed in either the middle or top tertiles of change for each of the three levels of WSS-ODA per capita received. Table 4 displays the ranges of change in population access to improved water and sanitation. The unadjusted odds ratios are presented alongside odds ratios adjusted for area, GDP, and per capita government health expenditure for 2006.

**Table 3:** Association between per capita WSS-ODA on the change in access to improved water and sanitation

Per capita WSS-ODA		OR of achieving top two tertiles of increased water access (95% CI)				OR of achieving top two tertiles of increased sanitation access (95% CI)			
**Year**	**Range ($USD)**	**Unadjusted**		**Adjusted^†^**		**Unadjusted**		**Adjusted^†^**	
		
2002	< 0.16	1.0	(reference)	1.0	(reference)	1.0	(reference)	1.0	(reference)
	0.16-0.52	1.55	(0.43-5.58)	1.91	(0.44-8.20)	0.58	(0.16-2.13)	1.43	(0.30-6.70)
	> 0.52	6.85*	(1.57-29.93)	8.50*	(1.73-41.64)	2.46	(0.60-10.03)	5.26*	(1.02-27.14)
									
2003	< 0.21	1.0	(reference)	1.0	(reference)	1.0	(reference)	1.0	(reference)
	0.21-0.69	1.08	(0.30-3.85)	1.35	(0.28-6.65)	0.40	(0.11-1.49)	1.61	(0.29-9.03)
	> 0.69	3.84	(0.98-14.98)	4.41*	(1.01-19.26)	1.28	(0.34-4.84)	2.78	(0.59-13.08)
									
2004	< 0.24	1.0	(reference)	1.0	(reference)	1.0	(reference)	1.0	(reference)
	0.24-0.72	10.55*	(2.41-46.15)	32.69*	(4.80-222.4)	0.83	(0.22-3.03)	2.59	(0.52-12.94)
	> 0.73	10.55*	(2.46-45.25)	18.15*	(3.46-95.21)	2.22	(0.60-8.12)	3.33	(0.78-14.21)
									
2005	< 0.19	1.0	(reference)	1.0	(reference)	1.0	(reference)	1.0	(reference)
	0.19-0.97	2.44	(0.67-8.90)	3.91	(0.89-17.17)	0.87	(0.24-3.11)	3.63	(0.74-17.94)
	> 0.97	3.86	(0.99-14.99)	4.54*	(1.05-19.58)	1.53	(0.41-5.74)	3.13	(0.66-14.72)
									
2006	< 0.36	1.0	(reference)	1.0	(reference)	1.0	(reference)	1.0	(reference)
	0.36-1.15	5.60*	(1.43-22.01)	8.38*	(1.82-38.69)	1.55	(0.43-5.59)	2.51	(0.56-11.16)
	> 1.15	6.63*	(1.60-27.46)	9.36*	(1.95-44.91)	2.06	(0.54-7.80)	3.39	(0.72-15.93)
									
2002-2006	< 1.54	1.0	(reference)	1.0	(reference)	1.0	(reference)	1.0	(reference)
	1.54-4.32	2.45	(0.67-8.97)	3.88	(0.85-17.71)	0.51	(0.14-1.85)	2.11	(0.44-10.19)
	> 4.32	6.65*	(1.64-26.87)	8.01*	(1.79-35.90)	2.30	(0.61-8.73)	3.70	(0.82-16.72)

*: Significant at the alpha = 0.05 level

OR: Odds ratios

CI: Confidence Interval

†: Adjusted for land area, Gross Domestic Product ($PPP), and per capita government health expenditure 2006

**Table 4:** Tertile ranges for relative change (2006 vs. 2000) in population access to improved water and sanitation

	Tertile level	Relative Change in population access (%)
Water	Lowest	-7.0 to 2.3
	Middle	2.4 to 8.5
	Highest	11.1 to 71.0
		
Sanitation	Lowest	-20.8 to 3.2
	Middle	3.7 to 14.8
	Highest	17.9 to 118.2

Significant odds ratios for water access and WSS-ODA per capita were observed for all years in the adjusted model, ranging from 4.4 (2003) to 32.7 (2004). Most odds ratios were not significant for sanitation and WSS-ODA per capita, with the exception of the adjusted model for 2002 (see Tables 3 and 4).

### Access and child health

Table 5 summarizes the odds of increasing access to safe water and sanitation by the amount observed in either the middle or top tertiles of change for each of the three levels of reduction in child mortality. Unadjusted odds ratios were presented alongside odds ratios adjusted for area, GDP, and per capita government health expenditure. Though not apparent in the unadjusted odds ratios, accounting for potential confounders uncovered an association between reductions in infant and child mortality and gains in population access to improved sanitation. No such association was found for water access. Reasons for this are discussed in the next section.

**Table 5:** Association between reductions in infant and child mortality and change^§^ in access to water and sanitation

% Reduction in mortality		OR of achieving top two tertiles of increased water access (95% CI)				OR of achieving top two tertiles of increased sanitation access (95% CI)			
**Indicator**	**Range (%)**	**Unadjusted**		**Adjusted**^†^		**Unadjusted**		**Adjusted^†^**	
		
IMR	<5.13	1.0	(reference)	1.0	(reference)	1.0	(reference)	1.0	(reference)
	5.13-11.82	1.55	(0.43-5.64)	1.56	(0.38-6.39)	1.09	(0.26-4.62)	1.80	(0.36-8.95)
	>11.82	1.32	(0.39-4.54)	1.39	(0.34-5.64)	3.41	(0.73-15.81)	8.00*	(1.30-49.34)
									
CMR	<5.46	1.0	(reference)	1.0	(reference)	1.0	(reference)	1.0	(reference)
	5.46-16.06	1.71	(0.47-6.22)	1.74	(0.42-7.21)	0.89	(0.21-3.79)	1.32	(0.26-6.61)
	>16.06	1.50	(0.44-5.17)	1.52	(0.37-6.21)	4.06	(0.86-19.18)	9.08*	(1.44-57.45)

§: Absolute (and not relative) change in percent access to water and sanitation

*: Significant at the alpha = 0.05 level

†: Adjusted for land area, Gross Domestic Product ($PPP), and per capita government health expenditure 2006

OR: Odds ratios, CI: Confidence Interval, CMR: Child mortality rate, IMR: Infant mortality rate

### Line equation for assistance and water access

We used the logistic procedure in SAS to compute the equation of the regression line for WSS-ODA per capita in 2004 and population access to improved water, adjusting for area, GDP, and government health expenditure. The equation of the line was as follows: Change in % population access to water = 3.8266 + 3.8457 * WSS-ODA per capita 2004.

Using this equation, it is estimated to cost $1.60 USD per capita to increase the number of people with access to improved water supply by 10% of the starting value. The immediate caution to this formula is that actual increases in coverage depend on how investment decisions are made and funds are administered. To make this formula more clear, consider an example of a population of one million people where 80% of the population currently has access to an improved water source. A 10% relative increase in access would be an 8% absolute increase. Thus, $1.6 million USD is theoretically required to increase population access to improved water from 80% to 88% for a population of 1 million.

## Discussion

Water and sanitation infrastructure substantially alters childhood mortality and morbidity [29]. However, the association between country level ODA and mortality has not been investigated. We have demonstrated that countries receiving the most WSS-ODA were 4-18 times more likely than countries in the lowest tertile of assistance to achieve greater gains in population access to improved water supply. We were unable to demonstrate consistent improvements in access to sanitation. Those countries with greatest gains in sanitation were 8-9 times more likely to have greater reductions in infant and child mortality.

Comparing the highest tertiles of WSS-ODA from 2002 to 2006, all of the adjusted odds ratios achieving change in the top two tertiles of change in population access to water were statistically significant and ranged from 4.41 times (1.01-19.26) in 2003 to 18.15 times (3.46-95.21) in 2004 more likely than the countries in the lowest tertile of WSS-ODA per capita. In general, countries falling in the highest tertile of per capita WSS-ODA are most likely to experience an increase in the relative percent of the population with access to improved water sources. For all years but 2004 and 2006, the countries falling within the middle tertile of WSS-ODA did not experience significantly higher odds of increasing population access to water than those in the lowest tertile. We propose this could be due to a lack of statistical power, or because of increasing population sizes, where WSS-ODA levels that fall below a certain threshold do not appear to increase access to coverage of water and sanitation services because the population is growing faster than additional services are being provided.

Despite trends of improved access to sanitation, most evaluations were statistically non-significant. It is unclear whether or not the lack of association is due to a true lack of association between WSS-ODA and sanitation, or whether or not, because of the higher complexity of sanitation systems, there is a lag period for the association to emerge. It may seem a paradox that overall, smaller relative gains were made in access to water compared to access to sanitation, yet WSS-ODA was only significantly related to the change in water access. A large factor in explaining this paradox is that the median baseline value for water access was much higher compared to that of sanitation (59% vs. 28%). Sanitation appears in some ways to be at odds with ODA and government health expenditures, as negative correlations were observed for both the sum of the total ODA per capita between 2002 and 2006 (-0.30, p = 0.041) and per capita government health expenditures in 2006 (-0.33, p = 0.025). Further analysis is required to explain the relationship between ODA and sanitation.

Interestingly, there was no significant correlation between total ODA per capita received by a country and any of the child health indicators. There was however a significant association between higher levels of increase in sanitation and reductions in infant and child mortality, with adjusted odds ratios of 8 and 9 times for the highest compared to the lowest tertiles, respectively. It is unknown why there is an apparent lack of association between this relationship and WSS-ODA. It may be due to ineffectiveness in investments, a weak capacity of the mandated national institutions, or perhaps due to success on behalf of local, non-internationally funded efforts. The higher odds of sanitation, as compared to water access, producing significant reductions in child mortality is consistent with the literature including a study by Fewtrell and co-workers [29-31], who showed that sanitation and hygiene have a greater impact in relative risk of acquiring diarrhea compared to water quality and water supply projects. And yet, at least for donors that do provide disaggregated WSS-ODA data, only 30% of funding goes to sanitation and hygiene efforts [32]. This highlights the need for decision-makers to be more intentional with allocating WSS-ODA towards sanitation projects.

While public health practitioners may consider water and sanitation to go hand in hand, this natural association must not be assumed in all cultural contexts [31]. Water, for example, is often interpreted as a broad community issue that contributes to the local economies in a variety of important ways, including employment based on clean water access, such as food sales. Sanitation, on the other hand, may be associated with cultural taboos, preventing local discussion of this important child health indicator [32]. Thus interventions must recognize the uniqueness in approach necessary to optimize maximum health benefits from water supply and sanitation and hygiene projects. Indeed, on an international level, sanitation is gaining more unique attention, as evidenced by the declaration by the United Nations of 2008 as the International Year of Sanitation. Similarly, the eThekwini Declaration was supported by 32 African ministers responsible for sanitation to ensure increased spending on sanitation [33]. The impact of these assurances need to be monitored. Currently the EU Water Initiative is working to provide a feasible strategy to disaggregate WSS-ODA data into aid for water supply, sanitation and hygiene, and water resources management [32]. When this data becomes available, a more thorough analysis of the relationship between water and sanitation-designated funding, and their respective impacts on health should be assessed.

As with any study, this research was bound by certain limitations. First, due to the nature of the research question dealing with only low-income countries, our sample size was relatively small, which constrained some steps in our statistical analysis. It was further constrained for analysis of health outcomes by the fact that diarrhoeal diseases account for an estimated 18% of child deaths [1]. Hence it is possible that with a larger number of countries, correlations and odds ratios of borderline significance would become significant.

Another limitation is that ecological studies are always to be interpreted with the understanding that cross-country comparisons cannot capture fully all of the unique socio-political, economic, cultural, and geographic factors that influence aid effectiveness in expanding water and sanitation infrastructure, and gains in child health made can be masked by other factors, such as increasing mortality from HIV/AIDS. Because of the scope of our research, we were unable to include an analysis of how conditions in conflict settings influence both ODA and its distribution and timeliness in expanding access to water and improved sanitation facilities. This is an important topic for future study.

As we approach 2015 and the world continues to labour to meet its commitment to the Millennium Development Goals, regular assessments should be carried out on the goals and their components. This study draws attention to the need for more research around ODA effectiveness in the expansion and maintenance of water and sanitation infrastructure. Despite the transfer of large amounts of ODA, many of the MDG targets are not expected to be met [13,23]. The G-8 summit in 2005 resulted in a commitment to double aid to Africa to help change the course of these projects, particularly in improving the delivery of government services and the building infrastructure for health, education, and water and sanitation [23]. Yet Thiele and colleagues found that proportions of total aid going to water and sanitation have decreased since the early 1990s, with the proportion designated to water and sanitation dropping from 4.9% to 3.9% and 1.1% to 0.8% in 2002-2004, respectively [34].

More research is needed to understand the seemingly paradoxical relationship between ODA and sanitation, how debt relief compares to grants and loans in proliferating water and sanitation infrastructure, what degree of public-private mixing in ownership and service provision is optimal for rapid expansion in certain resource-poor settings, and how public education can be used to compliment infrastructural expansion to produce synergistic benefits to child health. It would also be interesting to conduct an analysis determine the effectiveness of national allocations towards the water and sanitation sector.

Preparation for this study uncovered the absence of important data. To begin, our initial aim was to use diarrheal-specific mortality rates as our health outcome, since it is expected to have a stronger association with water and sanitation infrastructure than overall infant and child mortality rates. This indicator could not be employed since the percentage of deaths from diarrheal disease, as reported by the World Health Organization, was only reported for the year 2000. In addition to diarrheal mortality, we had desired to control for conflict, but could not because we were unable to find an appropriate conflict index scale.

Future research would benefit from the accessibility of sub-national level monitoring of progress in water and sanitation access as well as health surveillance. Since country-level data is often derived from census data, it is highly likely for many countries that district and even city-level data is available, but not accessible. We would strongly suggest that an international body, such as the UNICEF or the World Health Organization, solicit and make publicly available sub-national data, to help researchers avoid the ecological fallacy and be able to conduct precise and detailed inquiries.

## Competing interests

The authors declare that they have no competing interests.

## Authors' contributions

EP, MJB, MJ, and EM conceptualized the research question and developed the inclusion criteria, EP, MJB collected data on the variables. MJ, RB and MJB conceptualized and performed the statistical analysis. EP and MJB prepared the first draft of the manuscript. EP, MJB, MJ, EM, BJ and RB critiqued the draft, added text, and gave valuable input to refinement of the statistical analysis. Subsequent revisions were made by all authors. All authors reviewed the final draft and approved it for submission.
